# Opening up the Window into “Chemobrain”: A Neuroimaging Review

**DOI:** 10.3390/s130303169

**Published:** 2013-03-06

**Authors:** Carole S. Scherling, Andra Smith

**Affiliations:** 1 Memory and Aging Center, Neurology, UCSF, Sandler Neuroscience Center, 675 Nelson Rising Lane, San Francisco, CA 94158, USA; 2 School of Psychology, University of Ottawa, Vanier Hall, 136 Jean Jacques Lussier, Ottawa, ON K1N 6N5, Canada; E-Mail: asmith@uottawa.ca

**Keywords:** chemotherapy, cognitive impairment, chemobrain, neuroimaging, cancer survivorship issues

## Abstract

As more chemotherapy-treated cancer patients are reaching survivorship, side-effects such as cognitive impairment warrant research attention. The advent of neuroimaging has helped uncover a neural basis for these deficits. This paper offers a review of neuroimaging investigations in chemotherapy-treated adult cancer patients, discussing the benefits and limitations of each technique and study design. Additionally, despite the assumption given by the *chemobrain* label that chemotherapy is the only causative agent of these deficits, other factors will be considered. Suggestions are made on how to more comprehensively study these cognitive changes using imaging techniques, thereby promoting generalizability of the results to clinical applications. Continued investigations may yield better long-term quality of life outcomes by supporting patients' self-reports, and revealing brain regions being affected by chemotherapy.

## 1. Introduction

Recent advances in the diagnosis and treatment of cancers are allowing more patients to achieve complete physical recovery. With this positive trend has come growing concern about the long-term side-effects of treatment. Many patients report experiencing cognitive changes while undergoing chemotherapy. In some patients, these changes persist for years post-treatment, and can seriously affect their quality of life as well as that of family and friends. Patients have coined terms like *chemo fog* and *chemo brain* to refer to these changes, indicating their assumption that chemotherapy is the causative factor.

Results of objective neuropsychological assessments, however, do not always corroborate the deficits reported by patients, and thus such cognitive deficits have historically been dismissed as a consequence of stress alone. This can lead to further patient frustration as they do not feel justified in their complaints and continue to suffer without confirmation of their impairment. One must keep in mind that even subtle changes may have significant functional implications for persons confronting high cognitive demands. Over the last decade, several studies have been conducted in cancer patients to investigate the effects of chemotherapy on cognition, most finding that chemotherapy-treated patients perform more poorly on neurocognitive tests than non-exposed controls [1–20]. Even prospective studies [3,9,14,15,19,21–24], which additionally include pre-treatment baseline testing and closely matched controls, reveal subtle cognitive declines after chemotherapy exposure (of note, two studies reported no increase in the frequency of cognitive impairment in chemotherapy-treated breast cancer (BC) patients compared to healthy individuals [12,25]).

The estimated prevalence of cognitive deficits in chemotherapy treated populations is highly variable, with a range from 17% to 75% reported across studies [26]. Such variability makes it difficult to convince those outside the patient population of the reality of cognitive impairments found in cancer patients undergoing or having completed chemotherapy. The considerable variability in results from one study to the next are due to differences in key study design factors including: (1) sample size (many studies use only a small number of patients), (2) differences in the nature of the neuropsychological battery used (e.g., targeted tests or complete battery) resulting in differential sensitivity to subtle cognitive changes, (3) increased sensitivity of computerized testing in conjunction with pencil and paper assessments, (4) nature of the control group (example: healthy controls *vs.* non-chemotherapy patient group), (5) definition and/or criteria of cognitive impairment adopted, (6) effects of anesthesia on cognition for patients who also underwent surgery [27], (7) stress of cancer diagnosis and treatment, (8) existence of pre-treatment differences in cognition between BC patients and controls [28–31], (9) possible negative effects of endocrine treatment on cognition [23], and (10) data analysis methods used, in particular, whether impairment is defined at the group or individual level and, in the case of longitudinal studies, whether or not the analyses control for practice effects associated with repeated testing. These factors must be systematically controlled in future studies if progress is to be made in understanding the effects of cancer treatments on cognition. There are many reviews on neuropsychological assessments in cancer and chemotherapy-treated patients. For further and more detailed readings on the neuropsychological findings in chemotherapy-treated patients, please refer to the following most recent reviews [32–34].

The following review article will review the limited imaging research on chemotherapy-related cognitive impairments (or CRCI) in adult samples only. There will be a particular focus on women with BC since most CRCI imaging investigations are conducted in this population and investigations in brain tumour cancer populations present their own unique challenges. Both structural and functional imaging studies will be described and synthesized in separate summary tables, possible confounding variables to be considered in future studies will be discussed, as well as the need for better control groups and the challenge of translating current data to clinical practice.

## 2. Findings from Imaging Studies

Even with use of increasingly sophisticated performance-based assessments, there is still the concern that subtle chemotherapy-induced deficits are not being recognized or acknowledged. Additionally, the neural structures and/or circuits that are being affected by chemotherapy treatment are still relatively unknown. In an attempt to provide empirical evidence for chemotherapy-related CRCI, neuro-imaging tools are increasingly being used to examine the effects of chemotherapy on the brain and cognition [33,35,36]. Application of such tools could help uncover a neural basis for the subtle cognitive deficits in affected patients. However, there are only a handful of imaging studies that have examined the CRCI phenomenon and thus further brain imaging research is required. This has been acknowledged and a task force has been developed to discuss methodologies and application issues, including translational potential for the research to clinical practice [37,38]. At the March 2012 conference (Paris, France), the taskforce once again acknowledged the small number of studies in the field, yet the overlap in the regions reported to be affected in those studies were positively noted. However, many of the studies used a similar working memory task and therefore, these similarities between studies could be task specific. More discussion on this point will occur later in the paper.

### 2.1. Structural/Anatomical—Neural Changes

#### 2.1.1. Advantages

There are many techniques that can help uncover structural changes in the brain. The least invasive and best quality images are produced with magnetic resonance imaging (MRI). MRI can produce excellent two- or three-dimensional images of brain structure without the use of ionizing radiation or radioactive tracers. The detection mechanisms of this non-invasive technique are so precise that changes in neural structures (both cortical and subcortical) can be detected over time [39]. The main techniques applied to uncover tissue density (e.g., grey matter) or volume differences are manual segmentation protocols, automated methods (e.g., Freesurfer) and voxel-based morphometry (VBM) tools. Manual segmentation protocols are highly specific and there are validated sets of instructions concerning individual brain regions (e.g., hippocampus, amygdala) which require extensive training as well as inter-researcher reliability. Meanwhile, voxel-based morphometry (or VBM) techniques are a widely used automated technique that divides the brain into grey matter, white matter and cerebrospinal fluid (CSF) and can provide tissue density and/or volumes for whole brain or region of interest investigations.

Another technique requiring MRI technology is diffusion MRI, which allows further structural assessment of the brain- producing *in vivo* images of biological tissues weighted with the local micro-structural characteristics of water diffusion. Within this, there are two distinct classes of application. Diffusion weighted MRI, or DWI, provides information about damage to parts of the CNS. This technique measures the rate of water diffusion at a specific location and is best applied in a tissue where the diffusion rate typically appears to be the same when measured along any axis (e.g., grey matter). The second diffusion technique is called diffusion tensor MRI, or DTI, which provides information about connections among brain regions. This technique capitalizes on the magnitude and direction of diffusion through a particular internal structure. Water will diffuse more in the direction aligned with the fibers and less when perpendicular to the preferred direction. This technique is therefore best when examining tissues with an internal fibrous structure such as white matter tracts. An extended application to this last technique is the ability to derive neural tract direction information, with the assumption that the diffusion within each voxel is homogeneous and linear. A particular measure called fractional anisotropy (FA) assesses whether water movement in a certain voxel is unrestricted (water movement can occur along all axes) or restricted (water movement only occurs along 1 axis). A FA score closer to 1 means greater integrity of the tissue in that particular voxel (or restricted water movement) while a score closer to 0 means less integrity (or unrestricted water movement). Additionally, DTI techniques can be used to infer the white matter connectivity of the brain, otherwise known as tractography, which reveals brain region connections. Overall, both diffusion techniques can help uncover structural neural changes over time and possible differences between groups concerning neural tissue integrity. These are a measure of the efficiency of neural communication. (For a review on Diffusion MRI techniques, please refer to Johansen-Berg Behrens [40]).

#### 2.1.2. Review of Anatomical Findings

Structural brain changes have been associated with chemotherapy in cancer patients. Cerebral white matter is vulnerable to neurotoxins, such as chemotherapy agents, and use of MRI techniques has increased recognition of white matter changes related to drug intake (e.g., leukoencephalopathy in leukemia patients after taking immunosuppressive drugs). Furthermore, these white matter changes are strongly correlated with grey matter volume changes. Overall, it is thought that both white matter and grey matter changes can be reversible. The regions reported in the structural studies outlined below are also reported in Tables 1 and 2.

##### 2.1.2.1. Anatomical MRI

One of the first studies by Brown *et al.* [41] prospectively assessed white matter volume changes induced by high-dose chemotherapy using anatomical MRI techniques. Eight patients with advanced BC were scanned prior to beginning chemotherapy, with six patients receiving induction chemotherapy. All patients completed the chemotherapy regimen, but only four patients were available for the subsequent 1, 3, 6, 9 and 12 months post-chemotherapy follow-ups. Results indicated no structural abnormalities at baseline. However, three of the four patients scanned at the 3 month post-chemotherapy time point showed a progressive increase in abnormal white matter volume, with maximal volume changes ranging from 73–166 cm^3^. These volume changes subsequently stabilized from 6 to 12 months after chemotherapy completion. These investigators also conducted brain MR spectroscopy (MRS), a technique which is used to non-invasively measure metabolite levels in the brain. Results showed little to no change in metabolic ratios from baseline to post-chemotherapy. A small transient post-treatment decrease in *N*-acetylaspartate to creatine ratio (NAA/Cr) in one voxel in the right parieto-occipital lobe was suggested to be a reduction in neuronal integrity. Overall, the authors conclude that there are treatment-related white matter changes occurring with high-dose chemotherapy regimens, which progressively accumulate until around 6 months but are not accompanied by persistent neurologic symptoms. Minimal disturbance of the biological marker *N*-acetylaspartate, as indicated by the MRS, may partly explain this good neurological outcome. This study was the first to explore chemotherapy-related structural changes in the brain using a prospective design. Although the small patient sample resulted in limited power to test assumptions, this study was important because it led the way for subsequent anatomical studies of chemotherapy-related brain changes.

A second VBM assessment of BC patients was completed by Inagaki *et al.* [42]. They assessed volume differences between controls and patients at two different time points. Additionally, they controlled for the effects of having cancer itself. At year 1 (range 3–15 months post-chemotherapy), the scanned population included 51 adjuvant chemotherapy-treated BC survivors as well as 55 BC survivors who received surgical treatment only without chemotherapy. At year 3 (range of 27–39 months post-chemotherapy), they scanned 73 BC survivors who had received adjuvant chemotherapy (some participants also had scan 1, others were new study recruits), and 59 survivors who had received local therapy only. These VBM-processed brain images were referenced to images of healthy controls (year 1, n=55; year 3, n=37). At the one-year time point, the chemotherapy patients were found to have smaller volumes in key areas involved in cognitive processing, including grey matter losses in the right prefrontal cortex and para-hippocampal gyrus and white matter decreases in the bilateral middle frontal gyri, left para-hippocampal gyrus, left precuneus and right cingulate gyrus. Additionally, there was a strong positive correlation between volume loss and performance on tests of attention and memory (Wechsler Memory Scale Revised). Interestingly, these results were no longer evident at the 3-year follow-up scan. When pooling the cancer groups and comparing to healthy controls, there were no regional differences at the 1-year and 3-year scans. A serious weakness of this study was the failure to consider the effect of adjuvant endocrine therapy, given that anti-estrogen therapy has also been implicated in cognitive dysfunction in BC patients [6]. Another limitation is the lack of a pre-chemotherapy baseline, which makes it impossible to assess whether the observed abnormalities were present prior to treatment. Despite these limitations, the Inagaki study was one of the first to suggest, using an adequate sample, an effect of chemotherapy on brain structure. The areas showing differences are important for performance on everyday tasks of working memory and attention and help to support anecdotal reports of CRCIs.

Another study [43] applied the first prospective design to studying CRCI using structural imaging. The researchers recruited 17 BC patients who were receiving chemotherapy treatment, 12 BC patients who were not treated by chemotherapy, and 18 matched healthy controls. These patients completed high-resolution anatomical MRI at baseline (after surgery but before beginning treatment), 1 month after chemotherapy completion and 1 year later. VBM techniques were applied to quantify whole brain grey matter volume. Results indicated that there were no group differences at baseline. Both cancer groups showed grey matter decline at the 1 month mark compared to controls. Chemotherapy-treated patients revealed grey matter reductions in bilateral middle frontal gyri and left cerebellum, while non-chemotherapy patients had decreased volumes in the right cerebellum (suggestive of cancer itself or surgery itself changing the brain). At 1 month after chemotherapy, the treated group showed decreased grey matter in bilateral frontal, temporal and cerebellar regions as well as the right thalamus compared to baseline. In this same group, recovery of grey matter volume is suggested in the bilateral superior frontal, left middle frontal, right superior temporal and cerebellar regions at year 1. However, other areas did not recover grey matter volume at this time, including the right thalamus, right medial temporal lobe, left middle frontal gyrus, right precentral gyrus, right medial frontal and right superior frontal gyri as well as bilaterally in other regions of the cerebellum. A strength of this study's design was the control for the effect of surgery by using the “days between surgery date and baseline MRI scan” as a covariate in the group comparisons. Adding a covariate attenuated the significance of the results but they remained significant. The authors additionally applied other factors as covariates to reveal possible confounding effects but these were not significant. This indicates that some speculated confounding factors do not influence grey matter volume. However, much larger groups are essential to dissect the effects of any contributing factors being applied as covariates. Overall, this study is strong since it adopts the first prospective design of a structural assessment of the impact of chemotherapy on the brain. Additionally, the use of three groups (BC patients with chemotherapy, BC patients without chemotherapy and healthy matched controls) is an asset. This grants simultaneous comparisons of chemotherapy-treated patients to both healthy controls as well as BC patients without chemotherapy, the latter controlling for factors such as the presence of cancer, or stress of a cancer diagnosis. One must take into account that BC patients who did not receive chemotherapy underwent a different treatment which could present a confound itself, highlighting the importance of having a healthy control group in addition to cancer groups in such studies. Finally, the decreased grey matter volumes reported in this study overlap with those reported in previous structural studies, notably in the frontal and temporal regions [42]. Interestingly, this was the first study to report anatomical changes in cerebellar volumes, corroborating with studies revealing functional changes in this region [44].

A recent study was the largest investigation of neuroanatomical differences between chemotherapy-treated breast cancer survivors and healthy controls to date. In particular, Koppelmans *et al.* [45] extracted total and tissue segmented volumes from 184 survivors and 368 controls using VBM techniques to reveal global structural differences. Survivors revealed smaller total grey matter volume compared to controls and no white matter differences. Additionally the authors reported that survivors revealed smaller total brain volume, likely due to grey matter reduction. The only regional investigation involved hippocampal segmentation on 177 cancer patients compared to healthy controls using an in-house automated method. This targeted segmentation did not reveal any differences between groups. The obvious strength of this paper is the unusually large sample size. Another strength is the homogeneity of the population for treatment regimen and number of treatment cycles. Both groups are relatively well-matched on many factors, yet survivors have a higher education level compared to controls. This may be a bias to study participation since individuals with higher functioning are more likely to report even mild cognitive decline. One factor that was not reported was menopausal status. This factor would have been particularly interesting considering the possible neuroprotective nature of estrogen on grey matter volume, particularly in the hippocampus. Please refer to the estrogen section later in this review for more on this subject.

The most recent anatomical assessment in breast cancer patients is a prospective study using VBM techniques to examine 27 chemotherapy-treated BC patients, 29 non-chemotherapy-treated breast cancer patients and 28 matched healthy controls [46]. Chemotherapy-treated patients revealed reduced grey matter volume, following treatment, in the left middle frontal gyrus and the left superior frontal gyrus. Similarly, these patients showed significantly smaller grey matter volume in the left middle frontal gyrus when compared to healthy controls post-treatment. These results were accompanied by increased Behavior Rating Inventory of Executive Function for adults (BRIEF) scores in treated patients over time indicating more self-reported cognitive impairment, particularly with the ability to initiate problem-solving or activity. When these domain scores were considered as a covariate in the post-chemotherapy analysis, a negative correlation was observed indicating that reduced density in the left middle frontal gyrus is associated with higher levels of cognitive complaints. The authors also innovatively investigated a relationship between grey matter and executive functioning changes in relation to APOE E4 genetic status. No significant results were observed. However, it was noted that a greater percentage of chemotherapy-treated patients were E4 allele carriers (grouping patients with both 1 or both E4 alleles copies) compared to non-chemotherapy patients and healthy controls. Overall, this paper supports earlier findings from the same laboratory [43] of frontal lobe grey matter decreases after chemotherapy. As well, novel consideration of APOE E4 in the model attempted to uncover an individual risk factor that could predispose certain patients to more significant side effects of treatment. This type of pioneering investigation is required to advance research in this field.

Another VBM study with a novel approach to data analysis was the first to apply graph theoretical analysis to assess grey matter structural networks in 37 BC patients with a history of chemotherapy-treatment compared to 38 healthy controls [47]. A description of these analyses is beyond the scope of this review, however, can be read in the original manuscript [47]. Very briefly, this paper demonstrates changes in large scale brain network properties in BC survivors who received chemotherapy treatment. There is a more randomized configuration in patient brain networks compared to controls, translating into weaker regional connectivity and disrupted global neural organization. These results support previous VBM findings of diffuse anatomical atrophy after treatment. As well, results suggest compensatory mechanisms, which have been suggested by fMRI research (a discussion about these fMRI studies takes place below). The cross-sectional nature of this paper obviously makes it difficult to ascertain if these differences were present at baseline or an effect of treatment. As well, the rather large range of time since treatment for the breast cancer patients may cause some blending of short term and long term effects. Also, there is little data and discussion concerning demographic and neuropsychological states of each group which would be beneficial to compare these novel findings to more traditional measures used in this population. Yet these limitations are out-weighed by the novelty of this kind of investigation and the authors aptly mention the need for prospective studies using this technique.

##### 2.1.2.2. Diffusion MRI

Three studies have investigated cognitive impairment and brain connectivity in a chemotherapy-treated population using DTI. One study [48] measured white matter integrity (FA scores) in the genu and splenium of the corpus callosum. They recruited 10 chemotherapy-treated BC patients who reported cognitive problems, although unfortunately complaints were not formally assessed. Patients were enrolled in the study for an average of 22 months (range: 3–32 months) after therapy completion. They also scanned nine healthy, age- and education-matched women as controls. Participants completed a digit symbol test which assessed processing speed. Results showed slower processing speed in chemotherapy-treated participants. Chemotherapy treatment was also correlated with lower FA scores in the genu of the corpus callosum, indicating that adjuvant chemotherapy affects white matter integrity in this area. This area of the brain is important for communication between the hemispheres and may well explain the reduced processing speed reported in the chemotherapy-treated patients. Moreover, this decreased white matter integrity was significantly correlated to cognitive deficits reported by chemotherapy-treated BC patients. The BC patients in this study were all on anti-estrogen agents at the time of scanning but, like the anatomical studies above, the effects of endocrine treatment were not considered in the analysis and there was no pre-chemotherapy baseline. This study was, however, the first to indicate changes in white matter integrity following chemotherapy, as well as a correlation between decreased performance and this white matter damage.

A larger study [49] examined cerebral white matter integrity in 17 BC patients post-chemotherapy compared to 10 non-chemotherapy treated BC patients and 18 healthy matched controls. These researchers used DTI in combination with comprehensive neuropsychological testing and a self-report questionnaire concerning cognitive failure. The authors applied FA techniques but additionally measured mean diffusivity (MD), which quantifies water diffusion within a tissue and is affected by the cellular size, shape and integrity of that tissue [50]. MD values decline with increasing tissue barriers, such as cell membranes and myelin sheaths [51]. Water runs into less obstruction with edema, demyelination and axonal loss [52,53] and can travel further, thus a larger value is indicative of more free water and decreased tissue integrity. Deprez *et al*. [49] reported that, compared to non-chemotherapy patients and healthy controls, chemotherapy-treated BC patients showed decreased FA in frontal and temporal white matter tracts, as well as increased MD in frontal white matter. A voxel-based correlation analysis between FA scores and individual neuropsychological test scores revealed significant correlations between neuropsychological tests (attention and processing speed) and FA scores in the temporal and parietal white matter tracts. Furthermore, self-report cognitive failure questionnaire (CFQ) scores were also negatively correlated with frontal and parietal white matter FA scores. Overall, chemotherapy-treated BC patients performed worse on the neuropsychological tests than the other two groups, with seven of these patients classified as clinically impaired on follow-up analysis. Compared to controls, these “impaired” chemotherapy patients had lower FA values than their “unimpaired” chemotherapy-treated counterparts. Due to the limitations of their study design, this group of researchers performed a further longitudinal study whereby pre-chemotherapy and post-chemotherapy DTI, neuropsychology, depression and intelligence data was collected and reported [54]. Data from 34 premenopausal women with early-stage BC were compared with two control groups, one with 16 BC patients not exposed to chemotherapy and the other 19 age-matched healthy controls, before and 3-4 months after treatment. Results showed that white matter organization, particularly in the frontal, parietal and occipital white matter tracts, was negatively impacted by chemotherapy and that this correlated strongly with cognitive functioning scores. Like Abraham *et al.* [48], these authors suggest that micro-structural white matter changes or abnormalities may underlie reported cognitive dysfunctions found in chemotherapy-treated cancer patients.

de Ruiter *et al.* [55] published a study that used multiple techniques, including measures of VBM as well as diffusion. In addition to FA and MD score, they considered radial diffusivity (RD) which had not been investigated to date. Seventeen long-term BC survivors (mean = 9.5 years post-chemotherapy treatment) completed neuropsychological testing and multimodal neuroimaging sessions, and their data was compared to 15 survivors who did not receive chemotherapy. Chemotherapy-treated patients revealed more subjective cognitive complaints and a significantly poorer performance in the word fluency professions test. As well, treated patients made more errors during a Flanker task and a Tower of London task, both administered in an fMRI assessment presented in an earlier paper [56]. The authors skillfully compared the anatomical results from this paper to functional assessments, the latter discussed in the fMRI section below. First, grey matter VBM analysis revealed decreased density in left parietal and occipital cortices, as well as bilateral precuneus and cerebellum in chemotherapy patients compared to non-chemotherapy controls. When the reduced grey matter maps were overlaid on the hypoactivation fMRI maps acquired during a paired association memory test, there was overlap in the left lateral posterior parietal cortex. Second, three DTI assessments revealed possible chemotherapy-related side effects on white matter tracts post-chemotherapy. FA was decreased in the left corona radiata, external capsule, stratum as well as bilaterally in the thalamic radiation. Larger MD scores were shown in bilateral internal capsule, posterior thalamic radiation, stratum, corona radiata, superior longitudinal fasciculus, and the corpus callosum. Increased RD was revealed in the bilateral posterior thalamic radiation, left corona radiata, left external capsule, left sagittal stratum, and left internal capsule. Overlayed MD and RD maps on fMRI maps uncovered decreased white matter integrity located adjacent to areas showing hypoactivation of the lateral posterior parietal cortex during the encoding phase of the paired association memory test. Third, a 1H-MRS assessment revealed decreased NAA/Cr ratio for chemotherapy treated patient compared to controls. A negative correlation was found between NAA/Cr ratios and both MD and RD scores in chemotherapy group. In particular, MD scores negatively correlated with NAA/Cr in the left centrum semiovale as well as white matter regions in the bilateral corona radiata, superior longitudinal fasciculus and left internal capsule. Overall, this paper is the first to show anatomical repercussions of adjuvant chemotherapy-treatment in long-term breast cancer survivors using a combination of imaging and neuropsychological assessments. These results support the trend to include both structural and functional assessments in the study of CRCIs. This multimodal approach demonstrating anatomical changes due to chemotherapy, in relation to biological markers as well as functional and psychological capacities is a major strength of this particular study. This is the most comprehensive assessment to date in the study of CRCIs, a study design that would be particularly interesting to apply in a prospective study. Another strength of this study is the homogeneity of the chemotherapy-treated group concerning time since treatment. This is notable since many other studies of CRCIs have a patient population with a rather large range of times since treatment. This limited range increases confidence in the results truly representing side-effects of chemotherapy in long-term survivors.

#### 2.1.3. Use of Functional Neural Imaging

Anatomical assessments of the brain using MRI can indicate structural neural changes over time, as well as indicate differences between groups. As observed with the above mentioned study, however, combining structural MRI with functional imaging techniques is a more ideal approach to studying CRCIs.

### 2.2. Functional Neural Changes

#### 2.2.1. Advantages

Functional neuroimaging provides insight into the working brain. Several techniques can be used to obtain information about neural activity, for example, electroencephalography (EEG), positron emission tomography (PET) and functional magnetic resonance imaging (fMRI).

EEG is an imaging technique that is used to measure electric fields in the brain with use of electrodes carefully placed on the scalp. Whenever there is electrical activity in brain regions near the surface, this can be recorded by the electrodes. The P300 is a brain wave measured as an event-related potential (ERP), elicited by infrequent task-relevant stimuli, dependent on the participant's reaction to the stimuli and most strongly obtained by electrodes at the central vertex (border of the frontalparietal lobes; or Cz scalp location) [57,58]. The magnitude, timing and topography of an ERP are metrics of cognitive function. Another example is the N100 that is elicited by an unpredictable stimulus in the absence of task demands. EEG offers high temporal resolution (on the order of milliseconds) despite poor spatial resolution.

PET uses radioactively-labeled and metabolically-active chemicals (administered intravenously) to view metabolically active brain regions during a cognitively engaging task. The emissions from these radioactive chemicals are detected by the sensors in the scanner and then this data is computer processed to reveal their distribution throughout the brain. The most commonly used ligand to examine neurotransmitter activity is a form a glucose, called fludeoxyglucose (or FDG). The greatest benefit of using PET is the ability to show both blood flow changes as well as brain tissue metabolism (in particular both oxygen and glucose) in a “working brain”. As well, it is particularly useful in cases where damage is diffuse, with few apparent changes to gross brain volume and structure.

fMRI techniques also provide a window into the working brain as it is a non-invasive neuroimaging technique that localizes brain function in response to motor, sensory or cognitive tasks. The basis for fMRI is that increased neural activity in a region is accompanied by a substantial increase in local blood flow rich in oxygen (leading to a reduction of deoxyhemoglobin), which results in an increase of magnetic resonance (MR) signal intensity. This is called the blood oxygen level dependent (BOLD) effect [59,60] allowing for safe brain imaging with no exposure to ionizing radiation (as with PET). This technique offers excellent spatial resolution (2–3 millimeters) and moderately good temporal resolution (slower than EEG but faster than PET).

#### 2.2.2. Review of Functional Findings

In addition to the small number of structural imaging studies, a limited number of functional imaging studies has assessed neural changes in chemotherapy-treated cancer patients. The regions reported in both PET and fMRI studies outlined below are also reported in Tables 3 and 4.

##### 2.2.2.1. Electroencephalography (EEG)

Due to the high temporal resolution of EEG, this is a useful imaging technique for quantifying subtle differences in timing of neuronal firing. This electrical activity can be extracted and can partially explain subtle cognitive differences precipitated by chemotherapy treatment. One EEG laboratory has performed three separate ERP studies [60–62] with different BC comparison groups, including populations such as those non-exposed to chemotherapy but with radiation therapy and those who received chemotherapy-treatment (both high and low doses, differing regimens). Researchers first used an information processing task with three conditions with varying levels of difficulty measuring at the Cz scalp location and investigating the P300 wave [61,62]. There were no group differences in task accuracy, yet chemotherapy-treated patients revealed slower reaction times compared to non-chemotherapy controls when age was added as a covariate for one study [62]. Each of these two studies revealed chemotherapy treated BC patients having attenuated P300 waveforms compared to untreated controls. The authors suggested that the decreased P300 amplitude in chemotherapy patients indicates a decrease in the timing of mental processes, as smaller P300 amplitude is associated with more difficulty performing a task. In particular, high dose chemotherapy-treated patients revealed smaller P300 amplitude compared to non-treated controls yet standard dose patients did not [62]. This suggests an allocation problem concerning processing resources, particularly in patients receiving higher chemotherapy doses, which indicates that different regimens may have differential effects on the P300 component. In both studies, self-reported cognitive complaints did not correlate significantly with any of the behavioural or neuropsychological measures. Authors also applied an auditory oddball task [63] and investigated both the N100 and P300 waves. Differences continued to be revealed along the P300 component with chemotherapy-treated patients showing lower amplitude compared to non-chemotherapy control. Additionally, the surprising finding of CMF-treated patients (regimen: cyclophosphamide, methotrexate and fluorouracil) revealing shorter P300 latency compared to high-dose chemotherapy patients suggested that this could be the result of cognitive speeding due to decreased response inhibition capacities. Again, there were no task performance differences between groups. Conclusions drawn from these studies included that chemotherapy treatment can have effects on brain functioning and that a different regimen can cause differential effects. Limitations of each of these studies included the lack of a pre-treatment assessment, no comparisons to healthy controls, and high task complexity which may have led to stress and rumination, confounding the interpretation of results. Additionally, patients who received radiation may simply benefit more from task training sessions, which means that this group does not require as much energy expenditure to complete the task. These papers are the first to examine CRCIs using EEG technology. Additionally, while power is decreased with separate consideration of chemotherapy-treated patient groups, this highlighted the importance of considering type of treatment e.g., dose and content of regimen.

Finally, one prospective study using EEG methodologies is currently underway under the supervision of Halle Moore (Cleveland Clinic's solid tumor oncology unit). This study is comparing eight early-stage BC patients with matched controls using EEG tools and cognitive tests at three time points (before, during and after chemotherapy). While this participant number is very small, it will be the first prospective study using EEG tools investigating the chemotherapy-treated BC patient's brain.

##### 2.2.2.2. Positron Emission Tomography (PET)

One PET study of CRCI has been published to date. Silverman *et al.* [44] recruited 16 long-term chemotherapy treated BC survivors with memory problems (minimum 5 years post-chemotherapy; 11 patients also had tamoxifen) and eight non-chemotherapy-treated controls (five were BC survivors and three never had BC). A previously acquired standard reference group of 10 healthy controls who underwent a [F-18] fluorodeoxyglucose-PET study were also used. Investigators used [O-15] PET to study blood flow during a delayed-recall word memory task. Delayed recall questions activated a larger portion of the inferior frontal cortex in chemotherapy patients compared to untreated patients. Additionally, [F-18] fluorodeoxyglucose-PET was used to assess resting metabolism between chemotherapy treated patients and controls. Results demonstrated that chemotherapy patients showed lower resting brain metabolism, particularly in the left inferior frontal gyrus and in the contralateral cerebellum.

Furthermore, with more impaired performance on the delayed recall task, there was lower resting metabolism in the frontal cortex (the inferior frontal cortex is already less metabolically active under healthy circumstances). Also, basal ganglia metabolism was decreased in patients treated with both chemotherapy and tamoxifen. Overall, study results suggest that the frontal cortex required additional neural effort to successfully perform the task in the chemotherapy-treated subjects. While, this study does not have a pre-chemotherapy baseline assessment, it does consider the effects of combination treatment in the analysis (chemotherapy + tamoxifen). These alterations in brain activity in chemotherapy-treated patients are an indication that the brain is working differently, either through new neural recruitment or compensation, to accomplish the same performance [64–67]. This may alter subjective experience of cognitive efficiency, such that cognitive activity is more effortful and results in greater fatigue, and thus may underlie patients' cognitive complaints. This might explain why many chemotherapy-treated BC (and other cancer) patients fail to return to premorbid levels of social/occupational functioning despite excellent physical recovery, thus reducing quality of life.

##### 2.2.2.3. Functional Magnetic Resonance Imaging (fMRI)

###### Resting State

There is only one resting state paper investigating treatment effects in breast cancer patients to date. Resting state fMRI is interesting since it is task independent and less vulnerable to confounds related to task performance and individual aptitudes. As well, disruption to resting state activation patterns could help explain active state activity. Bruno *et al.* [68] investigated 34 BC survivors, previously treated with chemotherapy, and 27 healthy controls. Menopausal status was appropriately controlled for in all analyses, with more patients being post-menopausal. Patients showed decreased self-reported executive functioning and memory abilities compared to controls. In addition, patients displayed altered organization of global brain networks, indicated by decreased global clustering and disrupted network characteristics in the frontal, temporal and striatal regions. Therefore, the authors suggest disruptions in topological organization after chemotherapy treatment, which may lead to subjective cognitive complaints. This paper is particularly novel in the study of CRCIs since it is the only resting state fMRI paper. Understanding altered network organization after treatment could help better explain functional changes.

###### Active Tasks

An interesting case study by Ferguson *et al.* [69] involved a pair of monozygotic twins, only one of whom had received chemotherapy treatment for BC. While no significant performance differences were revealed on memory and executive functioning assessments, the chemotherapy-treated twin reported subjective cognitive complaints. Additionally, structural brain differences between the twins were assessed (anatomical MRI) and revealed that the chemotherapy treated twin had greater white matter lesion volume than the untreated sibling. Finally, fMRI during a working memory task revealed larger and more diffuse areas of activation in bilateral frontal and parietal regions in the chemotherapy treated twin. While the small population size is an obvious drawback, the differences between these two genetically identical individuals may reflect the effects of chemotherapy on one member of the pair. It also suggests that a larger scale twin study could reveal much about the neural underpinnings of CRCI, particularly if a pre-chemotherapy baseline assessment were included in the study design. Recruiting such a population would be ambitious.

Using fMRI during a working memory task, Saykin *et al.* [70] imaged fifteen chemotherapy-treated BC patients, seven local therapy only BC patients and seven healthy controls. Groups were matched for age, sex (all women), education, and estimated baseline intellectual ability. Working memory was assessed using an auditory N-back task with variable processing load requirements (0, 1, 2, and 3-back conditions). This was the first prospective functional neuroimaging study to establish an appropriate baseline by scanning participants prior to beginning chemotherapy or radiation treatments. Repeat scanning (time 2 scan) was conducted within one month of the completion of the chemotherapy treatment and at the same time point for non-chemotherapy exposed participants. The authors reported no group differences in task performance with the expected main effect of working memory load on performance across groups and time. All groups showed expected activation patterns for the most challenging load (3-back), with bilateral activations of frontal, parietal, and cerebellar regions (baseline and time 2 scan). However, fMRI results from the time 2 scan revealed that chemotherapy-treated patients showed increased activation in posterior frontal and parietal regions compared to controls and local therapy patients and less bilateral activity in more anterior frontal regions. Therefore, as seen in fMRI studies of other disorders associated with mild cognitive impairment, compensation may allow performance levels to be functionally maintained in spite of changes in brain activation. These findings illustrate that the cognitive changes associated with chemotherapy may be too subtle to reliably detect with standard neuropsychological assessments and underscore the importance of and potential for using fMRI to elucidate underlying neurobiological changes. In addition to highlighting the benefits of fMRI, this study [70] also demonstrated a practice effect whereby all groups showed improved 3-back task performance at the time 2 scan. Interestingly, this improvement over time correlated with increased bilateral prefrontal activations in all groups. Without a control group at both time points, this change in activation may have been wrongly attributed to the effects of chemotherapy. This emphasizes the importance of having a matched control group at each testing time point when examining a clinical population.

Another fMRI study [71] investigated verbal declarative memory in fourteen BC women (8 metastatic, 6 locally advanced) who had a history of adjuvant chemotherapy treatment (mean time since last treatment was 3.3 ± 3.3 years, range: 0.5–10.3 years). Patients were age and education-matched to healthy female controls. Participants completed salivary cortisol sampling and other questionnaires, both considered as distress measures, as well as an fMRI adapted verbal declarative memory encoding and recall task while in the scanner. Results indicated that there were no between group differences in any of the distress measures. While both patients and controls revealed similar response accuracy on the encoding component of the task, a trend revealed that patients had faster reaction times. Patients showed less activity in bilateral superior and middle frontal gyri and left postcentral gyrus. During the recall component, both groups revealed similar response accuracy yet BC patients had slower reaction times compared to controls. Patients showed greater activation in right superior temporal gyrus extending to bilateral fusiform, bilateral lingual gyri, left hippocampus, bilateral basal ganglia, right precentral gyrus, right superior and middle frontal gyri, bilateral inferior frontal gyrus, right cingulate gyrus, bilateral insula, bilateral parahippocampal gyrus, bilateral cuneus, bilateral precuneus, bilateral superior parietal lobe and bilateral cerebellum.

The authors suggested that quicker reaction times during encoding along with decreased prefrontal activations could be indicative of increased impulsivity in patients compared to controls. Also, while task accuracy is similar for both groups, patients require greater and more global neural effort than controls when attempting to recall task information, perhaps explaining the greater fatigue and frustration reported by patients post-treatment. The type of chemotherapy regimen was shown to contribute to differential patient verbal memory impairments; CMF treated patients showed lower prefrontal cortex activity during encoding compared to ACT treated patients (ACT regimen: adriamycin, cyclophosphamide, taxol/taxotere). This study was the first to examine verbal encoding and memory capacities of chemotherapy-treated patients in an fMRI setting, while also investigating distress variables. Additionally, it demonstrates differential neural activations with varying chemotherapy regimens, highlighting the importance of distinguishing between type of chemotherapy-treatment. Finally, the limitation of a small sample size in this cross sectional design is exacerbated by the rather large span of time since treatment completion, ranging from 6 months to 10.3 years after treatment. While the authors report no relationship between increased time since treatment and improved brain function, one must be careful to collapse short-term and long-term patients into one population, especially when a population is small and subject-driven effects are more probable. As well, many of the BC patients also underwent other cancer-related treatments such as radiation and tamoxifen administration which are additional confounding variables.

Another comprehensive fMRI study [56] examined 19 high dose adjuvant chemotherapy BC survivors (only 16 used in the fMRI analysis) and 15 non-chemotherapy treated BC survivors at 10 years after treatment completion. The authors measured performance on 16 neuropsychological tests as well as BOLD fMRI activation and task performance during two tasks, applying both age and estimated IQ as covariates. Overall, the chemotherapy group was more impaired than the control group on the neuropsychological tests. The chemotherapy group performed significantly worse on the word fluency proficiency test and showed a trend for slower reactions on the motor speed domain. Although not significant, these patients also performed numerically worse than controls on 13 of the remaining 14 tests. The first fMRI task was an adapted Tower of London task which extracts executive functioning involving planning capacities. The second task was a paired-associates task which assesses episodic memory. The fMRI data revealed that the chemotherapy group performed more poorly and quicker on the Tower of London task than controls. Additionally, the chemotherapy group showed hyporesponsiveness of the dorsolateral prefrontal cortex in an ROI analysis, as well as decreased activation in the bilateral posterior parietal cortex in a whole brain analysis. Additionally, this hyporesponsiveness was maintained in the chemotherapy treated group during the paired associates task compared to non-chemotherapy treated survivors in the parahippocampal gyrus in an ROI analysis as well as bilateral lateral posterior parietal cortex, left precuneus, right dorsal striatum, right inferior parietal cortex and left middle temporal gyrus in a whole brain analysis. The authors reported that the chemotherapy-treated group showed borderline significant impairment on recognition memory. Overall, the authors suggest that high dose adjuvant chemotherapy is associated with long-term cognitive impairments, linking dorsolateral prefrontal cortex hypoactivation with impaired planning behaviour observed in the chemotherapy-treated BC patients. Additionally, the authors indicate that quicker reaction times in patients, along with poorer accuracy could be an indication of increased impulsivity due to impaired attentional abilities. Both tasks, although very different paradigms, revealed parietal hypoactivation, which supports the idea that chemotherapy-treatment induces long-term effects on attentional abilities. This study is the first to examine fMRI data in a patient group 10 years after-treatment, thereby examining long-term negative effects of chemotherapy on cognitive function and linking it with regional brain activity. However, this is a cross-sectional design; a prospective long-term study could have provided more than a snapshot by dynamically showing how the chemotherapy-brain changes from baseline, as well as which neural activity is maintained, recuperated or continuously lost after treatment and over an extended period of time. Additionally, this study could have benefitted from comparisons with a matched healthy control group along with the non-chemotherapy survivor group to control for possible long-term effects related to differential treatments.

Kesler *et al.* [72] examined prefrontal executive abilities in 25 breast cancer survivors who had previously received surgery and chemotherapy treatment [mean time since treatment (SD) = 4.7 years (5.9)] compared to 19 surgery-only BC survivors [5.0 years (7.7)] and 18 healthy controls. All participants were female, matched for age, education level and menopausal status. The fMRI paradigm was a card-sorting task, requiring participants to determine implicit rules governing the computer's categorization of geometric figures. Results indicated that BC survivors overall demonstrated reduced activity compared to healthy controls in the left dorsolateral prefrontal cortex and the medial frontal gyrus. Chemotherapy-treated participants showed reduced activity in the left lateral prefrontal cortex, along with increased perseverative errors and slowed processing speed compared with both non-chemotherapy and healthy participants. As well, this reduction in activity was significantly correlated with higher disease severity and higher self-reports of cognitive deficits in the chemotherapy group. Also, increased cognitive deficits in the chemotherapy group correlated with older age and lower educational level. This paper presented a novel fMRI investigation in this population as it investigated activation during decision making processes rather than during working memory, a type of processing that has been repeatedly focused on. In addition, the balance of tamoxifen use in both of the cancer groups makes them more comparable. A limitation of this study is the obvious cross-sectional design as well as the large range of time passed since chemotherapy treatment in a small population. Other factors such as cancer stage at diagnosis and menopausal status were significantly discrepant between groups but since chemotherapy patients are more likely to have a higher disease stage and are more likely to have treatment-induced menopause, these are somewhat unavoidable. However, future studies should attempt to control for these factors, for example by collecting estrogen measurements and adding them as covariate in the analyses.

A more recent working memory study applied a verbal auditory n-back task in 16 chemotherapy-treated BC patients, 12 non-chemotherapy BC patients and 15 healthy controls [73]. This prospective study scanned chemotherapy patients at baseline (after surgery, before chemotherapy), 1 month and 1 year after treatment, yoking intervals for the two other groups. No performance differences on the fMRI task were revealed between groups, yet chemotherapy patients revealed a decrease in performance on high load task conditions at month 1 compared to baseline, with an improvement at year 1. All time points revealed differences in blood flow between patients and controls, as well as between chemotherapy treated patients and those patients not treated with chemotherapy. More specifically, at baseline the patients had significantly more activity in bilateral frontal lobes compared with controls and the chemotherapy treated patients had more activity than non-treated patients in the right inferior frontal lobe. Concurrently, the chemotherapy-treated patients revealed less activity in the left parietal lobule compared to healthy controls at all three scanning sessions. Both cancer groups showed decreased left inferior frontal activity immediately after chemotherapy compared to baseline. Chemotherapy patients revealed increased left thalamic and posterior middle temporal activity compared to controls and increased right cerebellar, left inferior precentral and left posterior middle temporal gyrus activity compared to non-chemotherapy patients. Activation in the middle frontal gyrus remained attenuated in the patients at the final imaging session. This paper was the first to present both pre- and post-chemotherapy effects concerning working memory, and aptly suggested this be conducted in larger cohorts.

A second prospective study [74], closely examined both pre- and post-chemotherapy effects while investigating verbal memory retrieval and related brain activity in 21 early-stage BC patients and 21 individually-matched healthy controls. Participants were scanned at baseline (prior to chemotherapy) and 1 month after treatment on an fMRI verbal recall task. Several results are of interest. First, fatigue, anxiety, depression, anger and confusion, as well as vigor were different between the groups at baseline and post-treatment. These factors were thus included as covariates in the fMRI model and fatigue, depression and anxiety significantly altered blood flow during the verbal memory task. Second, pre-chemotherapy, patients revealed increased right anterior cingulate activity, however this effect was decreased with the consideration of fatigue scores. Consideration of anxiety and depression also reduced activity in the left anterior cingulate. Post-treatment patients showed decreased activity in bilateral insula, right inferior orbitofrontal gyrus, right medial frontal gyrus, right superior and middle temporal gyri and left superior temporal pole. Fatigue scores largely removed these group differences, yet uncovered increased activity in patients in the left hippocampus and superior occipital gyrus. Third, when considering changes over time, controls did not show any differences in activation. Meanwhile patients had decreased activity from pre- to post-treatment in the bilateral insula and left inferior orbitofrontal cortex. In addition, consideration of days since surgery as a covariate eliminated this time effect. Fourth, regression analysis in patients revealed that a lower number of days since surgery was associated with hyporesponsiveness in bilateral insula and left inferior frontal cortex. Increased anxiety and fatigue scores both correlated with more activity in the right medial frontal gyrus, in addition to increased left hippocampal activity associated with higher fatigue scores. Major strengths of this study are the comprehensive prospective design, the unique assessment of verbal recall in this population, individually matched controls (compared to group matching) as well as the consideration of fatigue as a covariate. As well, consideration of days since surgery as a covariate, continues to reveal that there may be a window of vulnerability after surgery, whether due to anesthesia and/or stress which may predispose patients to structural and functional neurological changes during and following chemotherapy. This point will be discussed further in this review.

#### 2.2.3. Limitations of Functional Assessments

Although functional neuroimaging provides a view into the working brain, all of these techniques sacrifice either temporal or spatial resolution at the expense of the other. Ideally, a multi-modal imaging study of the CRCI would capitalize on the high sensitivity of both the spatial and temporal resolutions of two different but complimentary tools, for example, a combined EEG/fMRI study. Another important factor to mention concerning functional imaging studies in this field is the consistent use of small samples. Defining an appropriate sample size is difficult because it is dependent on the scale of the effect being measured and a prominent issue in this rather new field of study is that exact effects are still unknown. However, for standard neuroimaging studies, one must strive to assess no less than 20 participants [75]. More recent studies have begun to study larger groups.

## 3. Multifactorial Approach

### 3.1. Other Factors to Consider in the Study of CRCI

Research into the biological mechanisms underlying cognitive deficits in cancer patients must address a myriad of potential confounds and thus requires a multivariate, multi-technological approach. One possible factor mediating the impact of cancer and chemotherapy on cognition is stress.

#### 3.1.1. Stress and Glucocorticoids

Undoubtedly, a cancer diagnosis gives rise to intense chronic stress for many individuals, especially those who are subjected to prolonged treatments. Stress is routinely measured in CRCI studies through self-report, and this measure correlates highly with complaints of cognitive disturbances [3,6,9,11,14,17,76,77]. Stress is associated with increases in glucocorticoids (GCs), such as cortisol due to increased activity of the hypothalamic-pituitary-adrenal (HPA) axis. Increased levels of GCs, which can also result from therapeutic GC administration as part of the immunosuppressive therapy used in cancer treatment, have themselves been shown to affect brain anatomy [78–80] as well as memory functions such as working memory [81–83]. It is hypothesized that the hippocampus is particularly vulnerable to glucocorticoid-mediated excitotoxicity due to its high number of glucocorticoid (GC) receptors. In fact, smaller hippocampal volumes are seen in populations with higher stress reactivity profiles suggestive of higher circulating GCs [84–86]. One MRI study has shown transient smaller volumes in key cognitive regions (such as the hippocampus) in BC survivors treated with chemotherapy compared to those treated with surgery alone. These structural differences corresponded to lower scores in attention, visual memory, and concentration 1 year after chemotherapy [42].

It is not only the effect of GCs on structures like the hippocampus that may play a role in CRCI. The presence of higher circulating GCs expected during cancer diagnosis and treatment may also contribute to an increase in capillary permeability of the blood brain barrier [87] that could lead to augmented accessibility of chemotherapy agents to the brain. Contrary to previous thinking, antineoplastic agents (widely used in chemotherapy) appear to be able to pass through the blood brain barrier to penetrate the brain. This has been demonstrated in studies revealing higher than expected concentrations of antineoplastic agents in both brain tissue and cerebrospinal fluid (CSF) of treated patients [87,88]. Therefore, increased GC levels could potentially exacerbate the adverse effects of chemotherapy on the brain.

To date, a “flattened cortisol secretion pattern” has been reported in patient populations with BC [89,90]. Such a flattened pattern of cortisol secretion has been associated with impaired negative feedback of the HPA axis [91]. Meanwhile, significant group differences concerning diurnal cortisol reactivity between chemotherapy-treated patients and healthy controls are not always revealed [71]. Future research should sample diurnal cortisol and other distress variables in a larger sample, additionally controlling for other confounding variables such as surgery and type of chemotherapy regimens. Overall, given the possible role of GCs in cognitive disturbances related to cancer and cancer treatment, quantifiable measures of stress should be considered in future studies of CRCI. Similarly, suggestions for stress reduction may be useful as a means to assist patients with this potentially very difficult time in their lives. This may include yoga, meditation, deep breathing techniques or other forms of relaxation.

#### 3.1.2. Hemoglobin and Fatigue

Another factor postulated to underlie fatigue and cognitive symptoms in cancer patients is chemotherapy-induced anaemia, although empirical evidence on this relationship has been conflicting [3,6,9,10,13,16,17,92–98]. While it is usually assumed that cognitive disturbance is secondary to fatigue, it could also be the case that an increase in neural effort required to complete day to day tasks gives rise to fatigue. fMRI may yield some objective evidence on this issue, provided that good measures of fatigue and anaemia are included in functional neuroimaging studies of CRCI. One fMRI study considered fatigue scores as a covariate in their analysis of brain activity related to verbal working memory [74]. This factor not only influenced group differences but also uncovered additional differences, suggesting that patient fatigue is significantly impacting neural functioning.

#### 3.1.3. Estrogen

It has also been suggested that chemotherapy might affect cognition by lowering estrogen levels. Treatment-induced menopause has been identified as a risk factor for development of cognitive deficits in BC patients [9], and the deficits reported by chemotherapy-treated BC patients are similar to those reported after natural or surgical menopause [99]. The brain is rich in estrogen receptors, including areas involved in memory and executive functioning [96]. Estrogen has beneficial effects on cognitive function as it helps in neurogenesis, promotes synaptogenesis in the hippocampus CA1 region, and has a role in neuroprotection [2,100–102]. Decreased estrogen levels in healthy post-menopausal women have been associated with poorer verbal learning and memory [2,98,103]. Previous research suggests a “neuroprotective” role of estrogen in key memory structures such as the hippocampus [104]. Growing recognition of the effects of estrogen on cognition has led to examination of the cognitive impact of adjuvant hormonal therapies for BC. In cases where the breast tumour expresses estrogen and/or progesterone receptors, such hormonal therapies are administered for long periods of time (five years or longer). These therapies act either by selectively blocking estrogen receptors (selective estrogen receptor modulators, or SERMS such as tamoxifen) or by prohibiting estrogen synthesis (aromatase inhibitors such as arimidex). Some studies have shown that BC patients who received anti-estrogen therapy (in particular in tamoxifen-users) have increased memory problems, [105,106], but others find no such relationship [107–109]. While estrogen and cognition is extensively studied in an aging population and is applicable in a mostly older cancer patient group [110–113], more research focused on estrogen and cognition particularly in the chemotherapy-treated population, including younger patients, is needed (for more information, see [114] for a review on the cognitive effects of hormonal therapy in BC patients).

#### 3.1.4. Cytokines

Changes in cytokine activity related to cancer and cancer treatment is another potential mechanism for CRCI as such changes can result in fatigue and cognitive dysfunction. Cytokines are any number of small proteins that are secreted by the immune system and that interact with and modulate the activity of other cells in the immune system (*i.e.*, they are immunomodulatory proteins). Overproduction or inappropriate production of certain cytokines can result in disease, inflammation and tissue destruction. Cytokine activity in the hippocampus has been linked to a disruption in memory function, in particular memory consolidation [115]. Ongoing studies have reported that levels of multiple cytokines are elevated in all cancer patients, not just in chemotherapy-treated patients. These values were highest post-surgery and remained higher than controls for 6-60 months after diagnosis [116].

One study investigated the contributing role of inflammatory factors on both hippocampal anatomy and verbal memory performance in long-term female BC survivors. Using Freesurfer, Kesler *et al.* [117] found smaller left hippocampal volumes and decreased memory performance in 20 survivors (4.8 years post-treatment) and larger interleukin-6 (IL-6) and tumor necrosis factor (TNFα) peripheral serum levels compared to 23 healthy controls (group matched on age, education, global intelligence). In particular, decreased left hippocampal volumes in the BC group correlated with both lower IL-6 and higher TNFα levels. Similarly, decreased verbal memory performance correlated with smaller left hippocampal volume. Interestingly, elevated levels of TNFα were associated with less time post-treatment, indicating a relationship between inflammation and lingering effects of chemotherapy treatment. However, there was an important group discrepancy in menopausal status with 79% of the survivor sample being post-menopausal compared to 51% of healthy controls. Hormonal changes associated with menopausal status may be contributing to increases in self-reported cognitive deficits and decreased hippocampal volume. Another point of concern is large survivor sample heterogeneity as the analysis includes data from participants with a wide range of time since treatment. This study is the first to attempt to correlate peripheral cytokines to both anatomical changes in the post-treatment BC brain and memory abilities. Future studies should be conducted in larger samples, potentially attempting a prospective design, and examining regional differences between grey and white matter and/or subdividing the hippocampus into sections such as the head and tail.

#### 3.1.5. Cancer Itself

A major weakness of the majority of previous studies of chemotherapy-related cognitive dysfunction is the inclusion of data from post-chemotherapy patients only. A baseline assessment of a patient before treatment is essential in order to demonstrate that any changes in cognition are related to exposure to chemotherapy. More recent studies of CRCI have adopted this prospective approach and report cognitive deficiencies in cancer patients even before exposure to adjuvant treatment. Cimprich *et al.* [28] revealed pre-treatment cognitive impairments in 10 newly-diagnosed female BC patients compared to nine healthy controls (women with a negative mammogram in the last year). Participants completed a modified Verbal Working Memory task (VMT) during fMRI in order to examine selective attention and working memory. BC patients were less accurate and slower than controls in the high-demand condition of the VMT task. They also showed larger activation than controls in the right inferior frontal gyrus with more cognitive demand, as well as additional components of attention/working memory circuitry in both hemispheres.

Another aspect of the impact of cancer itself on brain functioning is pre-chemotherapy differences in disease severity. Kesler *et al.* [72] observed a negative correlation between disease stage and neural activity. More recently, Scherling *et al.* [29–31] have also demonstrated that prior to chemotherapy, BC patients have different patterns of neural activity and different volumes of certain brain structures compared to non-cancer controls. MRI/fMRI were used to image 23 women with BC before chemotherapy but following surgery to remove the breast tumour. Results varied depending on the covariates introduced, supporting the need for multifactorial research initiatives to study CRCI that include several of the potentially important variables mentioned here. Results also varied according to the fMRI task applied. The tasks performed examined novel executive domains, such as visuospatial working memory and response inhibition, not previously used in the study of CRCIs.

These studies are pivotal in highlighting the importance of baseline assessments of cancer patients before treatment. Without appropriate baseline assessments and scans, the effects of the disease itself could be mistaken for an effect of treatment. The above studies appropriately suggest that stress of a new cancer diagnosis, fatigue and potential sleep loss may also contribute to pre-treatment cognitive problems. These effects could additionally be exacerbated by the other confounding factors indicated above, including anxiety. Similarly, the type of imaging data analysis can impact what is reported as a significant effect. Recently, two prospective studies discussed above [73,74] have revealed baseline group differences, in addition to differences after chemotherapy treatment using region of interest analyses. Due to the large number of voxels considered in whole brain analyses, region of interest analyses can identify smaller effects by decreasing the number of multiple comparisons performed.

### 3.2. Appropriate Controls Are Required

Recruitment of chemotherapy-treated cancer patients in neuropsychological and imaging studies can be quite challenging and costly. Therefore, chemotherapy-treated study groups tend to be relatively small. However, attention must be paid to the fact that most of the studies examining chemotherapy-treated cancer patients occur in racially and ethnically homogeneous samples, which means that results from these studies may not be truly generalizable. Since controls are also matched to these patients, having the same background, a much larger and varied sample is required.

A control group matched to the patient population in question is essential for appropriate interpretation of study results. CRCI studies are beginning to adopt a prospective design by following the patient group before, during and after chemotherapy. In order to accurately control for potential practice effects on tasks over time, one must have a matched control group. In CRCI research, there is considerable debate on the best “control” population with the ideal as a “control” population that only differs on chemotherapy treatment. Most studies adopt a healthy, matched control group, as it is the easiest to form considering how difficult it is to match appropriate controls with chemotherapy-treated patients. However, using a healthy control group can present procedural difficulties. Although these patients have similar age, sex and education profiles, these healthy participants fail to match on many other variables. These groups will have different cancer-related cytokine activity, stressors related to cancer diagnosis and treatment, side effects of triggered early menopause and even potential effects of anesthesia from surgery.

Other studies have attempted to control for the “presence” of cancer by not only including a healthy control group, but also a cancer group receiving radiation. Participants in the latter group experience most of the same “cancer-related” biological and psychological effects, making the groups more homogeneous, but radiation effects themselves must be considered and how comparable these radiation patients are to the chemotherapy patients after treatment requires further investigation. Research has shown that fatigue is the major symptom associated with radiation treatment in BC patients [118] and fatigue is a confound factor which affects task performance. Further research into the differences between these two types of treatment in cancer patients outside of brain radiation and their potential differential effects on cognition is required. Therefore, admittedly these patients have the same cancer profile (e.g., stress, surgery, *etc*…) but more research is needed to truly identify if their different treatment may be a confound itself. Furthermore, previous studies have indicated that matching cancer patients (e.g., chemotherapy-treated and cancer controls) along one treatment is a taxing recruitment scenario. Yet, the reality for many cancer patients is the application of multiple treatment regimens, (e.g., surgery + chemotherapy + radiation or additional hormonal treatments) which the field has barely started to investigate. It is difficult to understand the impact of one treatment alone and thus multiple treatment regimens are even more challenging and present an even larger recruitment issue for appropriate controls.

Finally, another interesting control group, used in the Inagaki [42] study, is a cancer patient population who only underwent surgery, and no other therapies. This is a challenging group to recruit since a majority of patients who undergo cancer-related surgery will subsequently undergo another treatment (e.g., chemotherapy, radiation, hormonal). Yet, such a group could help identify possible confounding variables existing before chemotherapy treatment begins, especially those associated with anesthesia-related cognitive deficits. Previous literature has indicated that there may be cognitive decline after general anesthesia administration during surgery. One study [27] examined postoperative cognitive dysfunction in 1064 major non-cardiac surgery patients. They revealed that such deficits are common in adult populations of all ages, but in particular in the elderly (age 60 or older) who are additionally more likely to develop long-term deficits. Therefore, there are grounds to acknowledge that anesthesia administration has cognitive effects that could be misattributed to CRCI deficits in the future, especially since the majority of cancer patients are middle aged to elderly. However, while this population can help highlight effects such as stress of diagnosis and effects of anesthesia, finding such patients who additionally “match” the patients in the chemotherapy group would be very difficult.

Clearly, one of the major challenges in CRCI research is the choice of an appropriate control group. Recruiting healthy participants is a solid choice since it is already very difficult to find appropriate controls matched to chemotherapy-treated patients on many factors (for example: age, education, sex) and to find individuals in this increasingly aging population who do not have their own health issues (for example: history of mental illness, history of cancer, heart disease, *etc*…). Some studies have included not just one healthy control group but have additionally included a local-therapy group (surgery and radiation). Matching two different controls per chemotherapy-treated participant does create a stronger study. However, to really identify any effects would require a very large population. Additionally, individually matching patients to respective controls creates a much stronger study design but is not a commonly applied practice in imaging CRCI studies. To date, most have applied group-level matching on factors such as age, sex and education level. Very few studies, with the exception of [29–31,74] went one step beyond and meticulously matched each patient on additional factors such as menopausal status, marital status and IQ. The latter should be a common practice in this multifactorial field.

### 3.3. Consistency in Imaging Results Required

Currently, the interpretation of findings in CRCI studies, as well as the translation of results to clinical practice is restricted since there is still limited research on this topic and a lack of test-retest evidence. Anatomical studies do show some overlap in the regions reported as being significantly different in cancer patients after chemotherapy treatment and matched controls (see Table 1). In particular, bilateral frontal regions are most affected in the post-treatment patient population, which corresponds to the executive functioning deficits (such as problems with planning and working memory) that are consistently reported by patients. Therefore, it is no surprise that functional imaging investigations have largely focused on working memory (see Table 2 for brain regions and studies on this). Working memory tasks in studies of CRCIs consistently show neural activation differences between chemotherapy-treated patients and controls in frontal, temporal and parietal regions. However, the direction of this activity is varied since patients in some studies reveal increased activations while activity is lessened in other studies compared to controls and over time. This variability in activations related to memory may be due to many factors. First, while many of these tasks measure working memory, most have not applied the same task with the same parameters. Even a subtle variation within an fMRI task can cause significant differences in the overall study results (e.g., auditory *vs.* visual task stimuli, verbal *vs.* motor responses, longer *vs.* shorter task periods, longer *vs.* shorter rest periods, nature of the control condition, *etc*…). Therefore, although these tasks all measure some components within the working memory umbrella, they are essentially different from each other in many facets making it difficult to compare and generalize the results. Second, control groups are not consistent across studies. Third, there is variability existing within the patient population itself, related to such factors as days since surgery for the baseline scan or the time span between the end of treatment and scan dates in cross-sectional studies.

These three factors present a challenge in fMRI and PET studies. Even a slight variation in the task protocol or cohort selection can significantly modulate the results, thereby limiting generalizability and clinical application of the results. In order to better understand working memory and possible confounding variables in CRCI, future studies should adopt similar control groups and use comparable imaging tasks (e.g., N-back working memory task), while minimizing variability within the patient population on other factors unrelated to chemotherapy-treatment itself. Additionally, investigations of other executive functioning components, such as response inhibition, that are known to affect working memory should be carefully examined. Overall, “test-retest” reliability is an important component in any clinical population in order to compare and/or confirm existing results. This is currently lacking in CRCI imaging research, making it challenging to bridge current experimental data to the future possibilities of clinical practice.

## 4. Conclusions/Outlook

The cognitive deficits related to CRCI can persist for years and seriously affect the quality of life of the affected individual, as well as their family and friends. In light of the increasing survival rates for cancer patients, it is essential for patients and their health care providers to understand the potentially long-term adverse effects of therapy.

While neuropsychological assessments indicate that there are true impairments in chemotherapy-treated patients, the recent emergence of neuroimaging in this field has revealed further empirical evidence of a significant impact of chemotherapy on neural structure and function. Knowing that the chemo fog phenomenon is not unidimensional, a multifaceted approach is essential if a thorough understanding of the cognitive implications of cancer and cancer treatment is to be achieved. Such studies involve: a prospective design, carefully-chosen and doubled control groups (e.g., healthy controls as well as cancer patients undergoing radiation treatment), dose-dependent neuropsychological testing, multimodal neuroimaging (e.g., EEG, anatomical MRI and fMRI assessments), and biological markers sampling (e.g., haemoglobin, cortisol, estrogen). Such multifactorial and multimodal studies are most likely to clarify the validity, severity, prevalence, nature, and underlying mechanisms of chemotherapy-related cognitive impairments. This information should lead to improved support for afflicted patients, including the development of targeted medical as well as cognitive rehabilitative and behavioural therapies.

## Figures and Tables

**Table 1.** showing increased (↑) and decreased (↓) volume and/or tissue integrity in chemotherapy-treated patients compared to matched controls from papers reporting detailed regional differences.

**Study**	**Tool**	**GM and/or WM**	**Time Post-Chemo-Therapy**	**Frontal, limbic, Diencephalon, Midbrain**	**Temporal**	**Parietal**	**Occipital**	**Cerebellum**
Inagaki *et al.*, 2007	VBM	GM and WM	1 year	GM ↓ R prefrontal cortex	GM ↓ R para-hippocampus			
				WM ↓ B middle frontal gyri, R cingulate gyrus	WM ↓ L para-hippocampus	WM ↓ L precuneus		
			3 years	None	None	None		
Abraham *et al.*, 2008	FA	WM	22 months	↓ genu of corpus callosum				
McDonald *et al.*, 2010	VBM	GM	1 month	↓ B middle frontal gyri				↓ L cerebellum
Deprez *et al.*, 2010	FA	WM	4.8 months	↓ tissue integrity	↓ tissue integrity			
	MD			↓ tissue integrity				
Koppelman *et al.*, 2012	VBM	GM and WM	21.1 years	↓ whole brain grey matter and total brain volume (GM +WM)				
Kesler *et al.*, 2012 (discussed under the cytokine section)	Free-surfer	GM and WM	4.8 years		↓ L hippocampus			
McDonald *et al.*, 2012	VBM	GM	1 month	↓ left middle frontal gyrus				
de Ruiter *et al.*, 2012	VBM	GM	9.5 years			↓ L parietal, B precuneus	↓ L occipital cortex	↓ B cerebellum (most in L)
	FA	WM		↓ tissue integrity L corona radiata, L external capsule, L stratum, B thalamic radiation				
	MD			↓ tissue integrity B internal capsule, B posterior thalamic radiation, B statum, R internal capsule, B corona radiata, B superior longitudinal fasciculus, B corpus callosum (body and genu)				
	RD			↓ tissue integrity L corona radiata, L external capsule, L stratum (incl inferior longitudinal fasciculus and fronto-occipital fasciculus), Left internal capsule, B thalamic radiation				
Hosseini *et al.*, 2012	VBM	Network hubs in patients (Graph theory)		L anterior cingulate		R inferior parietal lobule, R supramarginal gyrus		

**Table 2.** showing increased (↑) and decreased (↓) volume and/or tissue integrity in chemotherapy-treated patients over time from papers reporting detailed regional differences.

**Study**	**Tool**	**GM and/or WM**	**Time Post-Chemotherapy**	**Frontal, Limbic, Diencephalon Midbrain**	**Temporal**	**Parietal**	**Occipital**	**Cerebellum**
McDonald *et al.*, 2010	VBM	GM	1 month *vs.* baseline	↓ L middle frontal gyrus, B superior frontal gyri, R medial frontal gyrus, R precentral gyrus, R thalamus	↓ R superior temporal gyrus, L parahippocampus,			↓ B cerebellum
			persisting at 1 year	↓ L middle frontal gyrus, R superior frontal gyrus, R medial frontal gyrus, R precentral gyrus, R thalamus				↓ B cerebellum
McDonald *et al.*, 2012	VBM	GM	1 month *vs*. baseline	↓ left middle frontal gyrus, L superior frontal gyrus				

a Note: the nature of the control group varies in each study, please see text body for more details. Abbreviations: GM = grey matter, WM = white matter, L = left, R = right, B = bilateral, Br.= Brodmann area, VBM= voxel-based morphometry, FA = fractional anisotropy, MD = Mean diffusivity, RD = radial diffusivity

**Table 3.** showing increased (↑) and decreased (↓) activity in chemotherapy-treated patients compared to matched controls during active PET and fMRI investigations.

**Study**	**Tool**	**Tasks**	**Frontal, Limbic, Diencephalon, Midbrain**	**Temporal**	**Parietal**	**Occipital**	**Cerebellum**
Silverman *et al.*, 2007	[O-15] PET	Delayed-recall word memory task	↑ L inferior frontal gyrus (near Br. 44 45), R superior frontal gyrus		↓L Br. 45 (lateral to precuneus), supramarginal gyrus	↓ R primary visual cortex	↑ R posterior lobe
	[F-18] FDG-PET	None (resting metabolism)	↓ L inferior gyrus				
Ferguson *et al.*, 2007	fMRI	Verbal N-back (working memory)	↑ B frontal widespread		↑ B widespread		
Saykin *et al.*, 2006	fMRI	Auditory N-back (working memory)	↑ B posterior frontal gyri ↓ B anterior frontal gyri		↑ B posterior parietal lobules		
Kesler *et al.*, 2009	fMRI	Verbal declarative memory- encoding	↓ L superior frontal gyrus, R superior frontal gyrus, B middle frontal gyri		↓ L postcentral gyrus		
		Verbal declarative memory- recall	↑ B basal ganglia, R precentral gyrus, R superior and middle frontal gyri, B inferior frontal gyri, R cingulate gyrus, B insula	↑ R superior temporal gyrus, B fusiform, L hippocampus, B parahippocampus	↑ B precuneus, B superior parietal lobule	↑ B lingual gyri, B cuneus	↑ B cerebellum
de Ruiter *et al.*, 2010	fMRI	Tower of London (planning)	↓ L dorsolateral prefrontal cortex		↓ B posterior parietal lobule		
		Paired-associates task (working memory)	↓ R dorsal striatum	↓ R parahippocampus, L middle temporal gyrus	↓ B lateral posterior parietal lobule, L precuneus, R inferior parietal lobule		
Kesler *et al.*, 2011	fMRI	Card-sorting (judgment, working memory)	↓ L caudal lateral prefrontal cortex				
McDonald *et al.*, 2012	fMRI	Auditory N-back BASELINE	↑ B frontal gyri, R inferior frontal gyrus		↓ L parietal lobule		
		1 MONTH			↓ L parietal lobule		
		1 YEAR	↑ B frontal gyri, R inferior frontal gyrus, B middle frontal gyrus ↓ L inferior frontal gyrus				
Lopez Zunini *et al.*, 2013	fMRI	Verbal recall (working memory)	↓ B insula, R inferior orbito-frontal gyrus, R medial frontal gyrus	↓ R superior and middle temporal gyrus, L superior temporal pole			

**Table 4.** showing increased (↑) and decreased (↓) activity in chemotherapy-treated patients over time from active-task fMRI papers reporting detailed regional differences.

**Study**	**Tool**	**GM and/or WM**	**Time post-chemotherapy**	**Frontal, limbic, diencephalon, midbrain**	**Temporal**	**Parietal**	**Occipital**	**Cerebellum**
McDonald *et al.*, 2012	fMRI	Auditory N-back	1 month *vs.* baseline (compared to controls)	↓ L inferior frontal cortex, B frontal regions ↑ L thalamus, L inferior precentral gyrus	↑ L posterior middle temporal gyrus, L middle temporal gyrus			↑ R cerebellum
			persisting at 1 year (compared to controls)	↓ B middle frontal gyrus				
Lopez Zunini *et al.*, 2013	fMRI	Verbal recall (working memory)	1 month *vs.* baseline (patients over time)	↓ B insula, L inferior orbitofrontal cortex				

b Note: the nature of the control group varies in each study, please see text body for more details. Abbreviations: L = left, R = right, B = bilateral, Br.= Brodmann area.
